# Liberty and injustice for all

**DOI:** 10.4103/2152-7806.64964

**Published:** 2010-06-30

**Authors:** Patrick J. Kelly

**Affiliations:** New York, NY, USA

“When there are too many policemen, there can be no liberty. When there are too many soldiers, there can be no peace. When there are too many lawyers, there can be no justice”.

Lin Yutang (1895-1976)

Our tort legal system should be about justice. But it's really about money and a lot of it changes hands. Nonetheless, only a relatively small amount of malpractice settlement winds up in the hands of the injured party - about 19%. Plaintiff's attorneys (33-50% of the settlement), expenses and court costs consume the rest. Over and above this we also contribute heavily to this medico-legal economy. “Our” insurance companies soak us with heavy premiums, build up huge cash reserves and overpay their executives. When it comes time to pay out, settlement is frequently cheaper than fighting the case. As I said, it's not about justice; it's about money.

Our malpractice insurance carriers provide and pay for our attorneys. But these lawyers know who signs their paychecks. Their primary allegiance is to the insurance carrier. We may think that they are defending us but they are really defending the insurance company. The insurance company's priority is to minimize losses and maximize annual profit. Preserving our professional reputations is not a priority.

The attorneys assigned to represent us are on retainer, get paid for “billable hours” and make the same income irrespective of whether they win the case, lose it or settle; some incentive! Plaintiff's attorneys, on the other hand, have a tremendous incentive. If they don't win, they get nothing. And they are also out the expenses necessary to bring a case to trial. These expenses can extend into the high six figures. For this reason, no prudent attorney will file a malpractice lawsuit without having a medical expert review the case and indicate that there has been an informed consent issue or, more usually, a “deviation from standard of practice”.

Expert witnesses are required to show that a deviation from standard of care occurred. Without expert witnesses there would be very little malpractice litigation because no sane lawyer would waste time and money preparing a medical malpractice case if he/she could not get some “expert” to say that a deviation from the standard of practice had, in fact, occurred. Regrettably, expert witnesses abound. They offer their services at legal conventions and advertise in law journals and on the Internet. Why? Money!

Expert witnesses for the plaintiff can receive thousands of dollars for their opinions and testimonies. Some neurosurgeons earn a comfortable living doing little but medical-legal work. Why many of them remain in our national organizations (which adds credibility to their testimonies) is beyond my comprehension. Why do we continue to provide these individuals with the guns to shoot us?

Although I applaud the fact that various committees in our national organizations screen complaints about expert witnesses in neurosurgical malpractice cases, the punishments meted out are “slaps on the wrist”. In my opinion: six or twelve month suspensions from the American Association of Neurological Surgeons (AANS ) for giving false testimony are inadequate. False testimony in exchange for a hefty expert witness fee denotes a basic character flaw. These people should be kicked out of our organizations. They should also be turned over to their local medical societies and have their medical licenses revoked. In cases where false testimony has been provided by neurosurgeons with an academic appointment, formal complaints from the AANS/CNS should be made to their universities.

Not all expert witnesses are dishonest. Some believe so strongly in their points of view that they are unwilling to consider alternatives and become crusaders (well-paid crusaders). But most of us know that standard of practice is open to differences of opinion and there is little Class I evidence to support much of what we do. So controversies in patient management are debated in the literature and at national meetings. These should be our forums for debate; not the courtroom.

When experts who can't agree on the “correct” management of even our most common pathologies square off in court, the cash registers of the medico-legal system start ringing. And when the “experts” having years of experience and familiar with the literature can't agree, what does the tort system do? It calls on 12 people pulled off the street, confuses them with days of conflicting testimony and lets them decide on the “correct” management. (The lawyers must be laughing up their sleeves!) Confused juries may vote with the side that entertains them better, the plaintiff that pulls at their heartstrings or lawyer with the most engaging personality.

Ideally, all of us would like to see major tort reform. But we have made little progress here. And why should we expect it? Many of our state and federal legislators are ex-attorneys who, like most politicians, want to get re-elected. This is done by making the majority of the electorate happy, kowtowing to the special interest groups that fund re-election campaigns and angering the smallest number of people possible. Unfortunately, physicians comprise only 0.2% of the U.S. population. The remaining 99.8% could care less about our “malpractice crisis”. Few re-elections will hinge on the “doctor vote”.

Sure, we have lobbying efforts that many of us (including me) support. But the Trial Lawyers' lobby makes the efforts of the American Medical Association (AMA) and other medical special interest groups seem paltry. Personally, I see no hope for reform until the malpractice situation becomes so severe that physicians quit or restrict services, access to care for the majority of US voters is severely compromised and the media gets on our bandwagon for a change. Or until we are all working for some hospital or, heaven forbid, the government.

